# Dose management in CT facility

**DOI:** 10.2349/biij.3.2.e43

**Published:** 2007-04-01

**Authors:** V Tsapaki, M Rehani

**Affiliations:** 1 Medical Physics Unit, Konstantopoulio Agia Olga Hospital, Athens, Greece; 2 International Atomic Energy Agency, Vienna, Austria

**Keywords:** Patient doses in computed tomography (CT), dose management in CT, dose optimisation in CT

## Abstract

Computed Tomography (CT) examinations have rapidly increased in number over the last few years due to recent advances such as the spiral, multidetector-row, CT fluoroscopy and Positron Emission Tomography (PET)-CT technology. This has resulted in a large increase in collective radiation dose as reported by many international organisations. It is also stated that frequently, image quality in CT exceeds the level required for confident diagnosis. This inevitably results in patient radiation doses that are higher than actually required, as also stressed by the US Food and Drug Administration (FDA) regarding the CT exposure of paediatric and small adult patients. However, the wide range in exposure parameters reported, as well as the different CT applications reveal the difficulty in standardising CT procedures. The purpose of this paper is to review the basic CT principles, outline the recent technological advances and their impact in patient radiation dose and finally suggest methods of radiation dose optimisation.

## INTRODUCTION

Computed Tomography (CT) has emerged as one of the most important imaging techniques of modern times. Starting with a bang in early 1970s with a great promise of exploring inner structure of the organs, it faced challenge from MRI in late 1970s and has emerged not only survivor but rather its clinical applications continue to increase [1-4]. The recent advances in CT such as multidetector-row technology, with sub-second acquisition and CT fluoroscopy have boosted CT applications, even more enabling interventional radiological (IR) procedures, which were traditionally performed with C-arm X-ray units. The continual increase in number of slices that can be scanned in one rotation of the X ray tube has brought multidetector computed tomography (MDCT) into dynamic imaging. MDCT is all set for playing an important role in angiography where it may be indicated as a replacement for conventional coronary angiography. The development of hybrid systems such as PET/CT, SPECT/CT and CT simulators in radiotherapy, and its incorporation in CT planning and dose delivery systems is moving CT from the domain of diagnostic radiology to other specialities.

Increasing applications mean increasing collective radiation dose to the population. But that is not bad as long as individual CT examination is clinically justified and doses are optimised to be not more than what is necessary. But experience shows that individual patient doses are increasing [1, 6-10]. In one of the reports from the United States, it was estimated that CT scanning accounts for more than 10 % of all radiological examinations and about two-thirds of the radiation dose to patients [11]. Regarding MDCT, one of the main problems in the initial systems, which were four detector scanners was the width of the X-ray beam in the z-direction. Since more than one row of detectors has to be exposed, a broader beam should be used compared to single row scanners so as to expose the outer detectors of the row, thus increasing the radiation dose. This problem is minimal in 16 detector scanners and above. Large variation in exposure parameters and patient doses even for a single CT examination have been reported [12-17]. It is noted that at specific exposure parameters, the radiation dose to the patient from various CT models can be totally different due to changing CT geometry and filtration. There is also growing realisation that very often CT image quality is much higher than actually required to produce accurate clinical diagnosis and a number of studies reported large dose reductions using modified exposure parameters [18-21]. Taking all these into consideration, as well as the continuous need to balance between the net benefits and the risks of using such a modality, various international organisations have published guidelines so as to standardize CT examinations and optimise radiation dose [22-23]. The European guidelines include image quality criteria for the most frequent CT examination, good imaging techniques and use of Diagnostic Reference Levels (DRLs) [22]. Since it is not appropriate to set dose limits on medical exposures, DRL is a useful quantity that facilitates the investigation of dose levels in various CT procedures and permits comparison of performance between different scanners and techniques [22]. DRLs provide the means to improve patient protection, if it is required, identify poor performance and monitor CT performance in periodic measurements [24-27]. The foregoing discussion reveals the need for proper management of radiation dose in a CT facility. This paper aims to review the situation with regards to patient exposure in CT examinations, and provide practical advice to manage the radiation dose while maintaining diagnostic confidence.

## CT DOSIMETRY METHODS

Patient exposure is quite different in CT than in conventional X-ray examinations, with the X-ray tube rotating around the patient producing thin slices of the irradiated body region. Therefore, dose calculation in CT is more complicated and requires the introduction of special dosimetric quantities such as the Computerised Tomography Dose Index (CTDI) and the weighted CTDI (CTDI_w_) for a single slice and the Dose Length Product (DLP) for a complete examination. These quantities are described in detail in the European Guidelines [22]. With the launch of multidetector-row scanners, volumetric CTDI (CTDI_vol_) was introduced in order to determine the dose in one rotation.

### Computerised Tomography Dose Index

CTDI is defined by the following equation:

(1) $$CTDI=\frac{1}{T}\int_{-\infty }^{+\infty }D(z)dz$$

T is the nominal slice thickness and D(z) is the dose profile along a line parallel to the Z-axis (tube rotation axis). CTDI integrates the radiation dose imparted within and beyond a single slice. It is measured using a specially designed pencil ionisation chamber with an active length of 100 mm both in free air at the centre of rotation (CTDI_air_) and within cylindrical polymethylacrylate (PMMA) phantoms of 16 and 32 cm in diameter, simulating the head and body of a patient, respectively. CTDI_c_ and CTDI_p_ are defined respectively as the CTDI values measured with a pencil chamber dosemeter positioned within the centre and in the periphery of the PMMA phantom. CTDI_p_ can thus be considered as a good approximation of the entrance surface dose (ESD).

CTDI_w _is used for approximating the average dose over a single slice in order to account for variations in dose values between the center and the periphery of the slice. It is defined by the following equation:

(2) $$CTDI_{w}=\frac{1}{3}CTDI_{c}+\frac{2}{3}CTDI_{p}$$

CTDI_p_ is the average of the four CTDI_p_ values measured in the periphery of the phantom (12, 3, 6 and 9 o’ clock).

CTDI_vol_ is introduced to determine the radiation dose in one tube rotation in multidetector-row scanners and allows for variations in exposure in the z direction when the pitch (pitch is the ratio of table feed in one rotation to slice collimation) is not equal to one (CTDI_vol_ = CTDI_w_ / pitch). In the case of a single slice spiral system, CTDI_vol_ is equal to CTDI_w_.

### Dose Length Product

DLP is used to calculate the dose for a series of slices or a complete examination and is defined by the following equation:

(3) $$DLP=\sum_{i}^{N}CTDI_{w}TN$$

i represents each one of the individual N scans of the examination that covers a length T of patient anatomy. Certain manufacturers display the DLP value in each patient examination.

### Effective Dose

The effective dose is a “dose” parameter that reflects the risk of a non-uniform exposure in terms of a whole body exposure. It is a concept used to normalise partial body irradiations relative to whole body irradiations to enable comparisons of risk [28]. The calculation of effective dose requires knowledge of the dose to specific sensitive organs within the body, which are typically obtained from Monte Carlo modeling of absorbed organ doses within mathematical anthropomorphic phantoms [29], and recently also voxel phantoms based on real humans. Effective dose is expressed in the units of milliSieverts (mSv), and can be compared to the effective dose from other sources of ionising radiation, such as that from background radiation level (e.g., radon, cosmic radiation, etc.), which is typically in the range of 1 to 3 mSv depending upon the location. The International Commission on Radiological Protection (ICRP) emphasises that effective dose is intended for use as a protection quantity on the basis of reference values and therefore should not be used for epidemiological evaluations, nor should it be used for any specific investigations of human exposure. Rather, absorbed dose should be used with the most appropriate biokinetic biological effectiveness and the risk factor data. The use of effective dose for assessing the exposure of patients has severe limitations. An effective dose can be of some value for comparing doses from different diagnostic and therapeutic procedures and for comparing the use of similar technologies and procedures in different hospitals and countries as well as from use of different technologies for the same medical examinations. For planning the exposure of patients and risk-benefit assessments, however, the equivalent dose or the absorbed dose to irradiated tissues is the more relevant quantity. It must be remembered that an effective dose, however, does not tell the complete story with regards to the potential effects of ionising radiation. Specific organs and tissues are known to be more radiosensitive than others. While this is reflected in effective dose, the absolute doses to specific organs or tissues is also important to consider.

CTDI can be measured using pencil ionisation chamber. If measurements are not possible, the Imaging Performance and Assessment of CT (ImPACT) Patient Dosimetry Program produced by the Medicines and Healthcare products Regulatory Agency (MHRA) can also be used. The program is freely available on the Internet and continuously updated to include data on the recently developed CT scanners [14]. It provides CTDI and DLP values for a wide range of CT scanners and exposure parameters used to perform a CT examination. Furthermore, the Electrotechnical Commission in 1999 recommended the display of CTDI value on the CT console [30]. Many manufacturers currently display CTDI_w_ and CTDI_vol_ values on operator console. In this way, radiation dose adjustments during modification of exposure parameters can be viewed on the console before irradiation. The dosimetric quantities chosen to determine DRLs are CTDI_w_ (CTDI_vol_ in the case of multidetector-row scanners) and DLP. DRL values are proposed by the European Commission [22] and the National Radiological Protection Board (NRPB) [31]. These values should not be used individually. They should be the tool so as to identify situations in which dose optimisation should be applied.

## EXPOSURE PARAMETERS AND CT DOSE

Choosing exposure parameters is a complex task and depends to a large extent on the anatomical region to be scanned, the size and the pathology of the patient. The chosen parameters should result in sufficient image quality so as to aid clinical diagnosis. The main problem in determining exposure parameters is image noise and its effect on image quality. Some parameters that are in control of operators are discussed below:

**kVp**: Most CT systems do not provide users with flexibility to adjust kilo voltage (kV) or kilo voltage peak (kVp) in a continuous manner but there are few discreet settings possible. Tube kVp determines the quality and quantity of radiation. The intensity of X ray beam is typically proportional to square of kVp applied to the tube. Thus even minor modifications in the tube potential value can result in significant changes in image noise and considerable change in radiation dose [3]. According to Kopp [32], most of the abdominal CT examinations can be done using 120 kVp and earn a 20% to 40% reduction in radiation dose compared to a value of 140 kVp. Furthermore, paediatric CT examinations can be successfully performed using 80 kVp resulting in sufficient image quality [33-35].

**mAs**: Another important parameter which greatly affects image quality and dose is the product of tube current and rotation time (mAs). Radiation dose, at fixed kVp and filteration, is linearly related to mAs, meaning that by reducing the mAs by half, the dose is also reduced by half. On the other hand, noise is inversely related to mAs. Therefore, the reduction by half of mAs will result in a 50 % increase in image noise. A lot of studies have investigated the modification of mAs as a means of reducing the radiation dose and results showed that it is an easy and straightforward way of CT dose optimisation [16-18]. Certain studies have presented results on individual modification of mAs with respect to patient weight and showed substantial reduction in radiation dose [16, 18, 36]. It should be noted, however, that mAs modification should be done carefully in certain examinations such as the abdominal CT. The reason is that the increase in image noise can greatly influence image quality, which is very important in organs like the liver and pancreas.

**Pitch**: Pitch is another important parameter for spiral and MDCT. By definition, pitch depends on collimation and table feed. Therefore, if the patient’s table moves faster this will increase pitch and consequently decrease the duration of patient exposure and reduce radiation dose. However, a faster moving table results in certain artefacts, which have great impact on image quality. According to Kalra [3], no marked difference in abdominal image quality was noted between scans obtained with pitch 1.5 and those with pitch 0.75 resulting in 50% reduction of radiation dose. As far as collimation is concerned, small values inevitably result in higher mAs and consequently higher dose so as to maintain image quality. Specifically for multidetector-row scanners one should be careful as there are two definitions available for pitch depending on whether single section collimation (pitch: p which is independent of the number of detector rows) or the total collimation of the detector array (volume pitch: p* which increases as the number of detector row increases) is chosen as reference. In scanners using the volume pitch, values are usually higher (in the order of 6) than in scanners using conventional definition of pitch (p).

**Scan length**: The extent of body length covered in scanning does not affect the CTDI value but certainly affects DLP. The scanning length for a particular type of CT examination can vary due to the pathology of the patient, the size of the patient, the experience of the user, or even the demographics of a country (height of the population). With the evolution of CT scanners (non helical machines are almost extinct in developed countries), and especially with the introduction of multidetector-row scanners and the dramatic reduction of rotation times to subsecond values, users are tempted to extend the region of interest beyond the one actually required. For all these reasons, CT protocols need to be established so as to limit irradiation only to the particular body region in investigation.

## INTERVENTIONAL CT PROCEDURES

The evolution of CT technology has facilitated the wider use of CT in Interventional Radiology (IR) procedures. They are performed either by using conventional CT equipment in which the catheter needle and the lesion are observed during consecutive CT slices (blind technique) or by using CT systems that are combined with a fluoroscopy unit. CT fluoroscopy works in low tube currents in the level of 50 mA. Images can be acquired with frame rates that can reach 12 images/second. A special footswitch is used to control the patient table movement. Usually a monitor would be placed inside the CT scanner room. An important advantage of conventional CT is that it does not involve exposure to the medical personnel as CT fluoroscopy does. On the other hand, repetition of one or more slices in the region of interest is inevitable resulting in increased levels of radiation dose to the patient. While data on patient doses from interventional procedures carried out in angiographic units are widely available, only two studies investigated the levels of patient radiation dose in conventional CT intervention reporting maximum skin doses in the range of 500 mGy to 1000 mGy [37, 38]. A larger number of studies exist for CT fluoroscopy [39-42].

## PET-CT

With the recent achievement of combining PET with CT, corresponding examinations can be done without moving the patient but just moving the patient table to reach the body region to be examined. The CT images are used for producing the attenuation correction maps for PET images. On the other hand, the PET-CT patient undergoes a CT examination regardless of having a similar examination in a conventional system, adding to the total patient dose. To overcome this problem, low dose CT is performed using thicker collimation and lower mAs. The low dose CT acquisition protocol does not significantly affect attenuation correction and anatomic delineation in PET [43, 44].

## PRACTICAL WAYS IF OPTIMISING RADIATION DOSE IN CT

### Justification

It is one of the ALARA (As Low As Reasonably Achievable) principles and it is the first rule of optimisation in any radiology department. Due to the fact that CT procedure is classified as a high radiation dose procedure [45], it is essential that it is requested by properly trained practitioners in close collaboration with the CT radiologist. International Basic Safety Standards (BSS) require that an examination should be carried out only in the case of a justifiable clinical indication [46]. In certain clinical situations, non-ionising techniques such as ultrasound or magnetic resonance imaging (MRI) could probably provide similar information without irradiating the patient. The establishment of standard protocols for the most frequent examinations will limit radiation dose only to the level really required. Furthermore, the repetition of a non-enhanced CT procedure using contrast material should be reconsidered. In most cases, a single contrast enhanced examination is sufficient and the non-contrast procedure could be eliminated such as in abdominal CT for the evaluation of the liver. It should be noted, that contrast material and multiphase enhancement studies are a common practice over the last few years irradiating the same part of the body even up to four times [47]. Due to all these reasons, the Royal College of Radiologists in United Kingdom has produced clinical guidelines, which are really helpful in justifying a CT examination [48]. As far as repetition of a CT scan is concerned, it must be stressed that a second CT in the same body region is in most cases not justified.

### Shielding of organs

Shielding should be used in sensitive groups such as children and young patients. Shielding of organs such as the thyroid, eye lens and breast, when they are not in the primary beam can result in 40% to 80% radiation dose reduction [49]. A reduction of 95% in radiation dose can be achieved by shielding the testes in abdominal procedures [50].

### Modification of exposure parameters

The most easy and straightforward way of reducing the dose in CT is to lower the mAs. This can have a significant effect in image quality but in some CT procedures such as chest and the pelvic exam, this degradation does not usually have an impact in clinical diagnosis [51, 52]. In abdominal procedures, however, large mAs reduction is not usually possible. In these situations, modification of mAs according to patient weight can provide an alternative to dose optimisation [18]. Aldrich found that image noise is highly correlated with patient weight and that an acceptable image quality is associated with a noise level of 4.5. He then developed a simple mAs prediction equation to optimise radiation dose for all patient weight categories. The International Atomic Energy Agency (IAEA), through a coordinated research project (CRP) that involved six countries and nine new technology CT scanners across the world investigated the potential for patient dose reduction while maintaining diagnostic confidence in routine chest and abdomen CT examinations in adult populations. The main objective of the project was to develop a simple and clear-cut methodology whereby users could determine exposure factors that could be applied to patients of different body weight, rather than depend on the current approach of using default values based upon standard sized patient. The results showed that patient weight can be an excellent predictor of the required dose for routine chest and abdomen CT and that a noise level of 10 provided acceptable image quality, but the value could be increased for larger patients. The project also developed recommendations of how to implement the results to any CT department. Special exposure factors should be used for children. Reduction of mAs during the IR technique using the conventional CT machine will greatly reduce patient dose especially in the region of the body, which is scanned repeatedly in the attempt to position the guidance needle.

Another straightforward way to optimise dose is to increase the pitch of the exam either by increasing table speed or decreasing collimation. The choice of pitch will depend on the clinical situation and pathology of the patient such as in the case of pulmonary nodules in which increase of pitch is not encouraged due to the resulting reduction in their detection.

### Limitation of scan length

In order to limit the region of the patient being irradiated, only radiologists properly trained in CT as well as radiation protection issues related to the CT technique should perform such procedures. Consideration should be given to program the examination protocol according to pathology. The large range in DLP values reported in the literature reveal the differences in technique followed in each CT department [16]. For example, some operators examine the upper abdomen in cases of hepatic and pancreatic disease, whereas others examine the whole abdomen, which also includes the pelvic region. According to Hidajat *et al*, many clinical studies have to be performed so as to gain consensus for the optimal length of examination [53].

### Use of anatomy-adapted tube current modulation

Tube current modulation is based on the idea that pixel noise on the image results from quantum noise in the different projections taken as the tube rotates around the patient [17]. The value of mAs is therefore changed during one rotation according to the patient anatomy in each projection. The idea is similar to the automatic exposure control system in the X-ray radiography equipment. In the projection with less attenuation from the patient, such as the posterior-anterior chest projection, less mAs can be used. In lateral projections in which attenuation from the patient can be high, the mAs can be increased accordingly.

### Filtration

X-ray filters are used in radiology for cutting off the X-rays that have lower energy and do not contribute to the image but only to the patient dose. There are studies in the literature that have investigated the use of various filters and their effect on dose reduction [53, 55]. According to these studies, bow-tie or beam shaping filters reduce radiation dose by 50% compared with conventional flat filters. Software noise reduction filters is an alternative, especially in high contrast examinations such as chest CT. Kalra used such filters for post-processing reduced radiation dose chest CT images and found improved levels of noise in the lung, mediastinum and chest walls with some small compromise in image sharpness and contrast [56].

### Diagnostic Reference Levels

CTDI and DLP measurements should be part of the dose optimisation programme in a CT department. Determination of local DRLs should be done using a sample of 10 standard-sized (70 kg) patients in each type of procedure and mean values of the results should be compared to DRLs set by professional bodies [22, 31]. In the case of local values being higher than internationally set DRLs, corrective action should be applied after detailed investigation and thorough revision so as to reduce patient doses if deemed necessary. The procedure should be repeated in certain time intervals as part of the established quality assurance program, or when new techniques or new equipment are introduced in the department.

### Optimisation of PET-CT dose

In most PET-CT examinations, the quality of CT does not need to be in the level of the diagnostic CT. The reason is that CT images are just used to produce the attenuation correction maps needed for PET images. Therefore, lower exposure factors could be used such as lowering the mAs to 70-90 mAs. It should be noted however, that in some situations artefacts are possible when using low mAs values due to photon starvation effects. CT optimisation should be done carefully so as not to produce artefacts to the images. When implants were present, artefacts will most probably be present, so attention should be drawn to the correct interpretation of PET-CT images. Another possible cause of artefact could be the use of iodine-based contrast materials. The reason is that, at PET energies (511 keV), the iodine attenuation coefficient is close to that of water and this can cause artefacts in PET images. In this case, inspecting both the CT and PET images could help overcome this problem [57, 58].

### Training of staff

It was recently found that the concept of CT radiation dose is not fully understood or appreciated [59]. Therefore, the requesting physicians must be adequately informed of all associated risks when requesting a CT scan and must know how to balance the benefits and possible risks. Furthermore, the radiologists must be familiar with modification techniques according to the patient’s clinical situation and not simply apply set clinical protocols.

## CONCLUSION

New technological improvements such as the multidetector-row CT and the PET-CT systems opened the field for new and wider applications. Dose data in the literature indicate that manufacturers are focussing their efforts towards improving image quality with reduced radiation dose compared to older generation equipment, recognising the fact that dose reduction has, in recent years, been an important issue for users. However, the new technology CT can be operated so easily and quickly compared to previous years and tempts operators to overuse the modality. It should be also stressed that CT cancer screening studies are steadily increasing with the introduction of multidetector-row scanners posing substantial risk for lung cancer development from yearly screening and lower risk for colon screening due to shorter screening intervals [60]. In these cases, radiation dose optimisation is essential because it results in a 5 to 10 reduction factor. It is also widely recognised that the exposure factors applied are usually higher than actually required for getting an image with diagnostic confidence. Continuing development in scanner technology will no doubt further extend the indications for and scope of CT examinations. Ongoing clinical studies monitoring the associated patient dose can play a role in achieving excellent imaging at a reasonable patient dose. Initial steps for dose optimisation could include:

1. Clear justification of examination
2. Avoid repetition of examination
3. Use of tube current modulation
4. If the clinical situation and the pathology of the patient permit, increase the pitch of examination.
5. In the case of scanners that do not have tube current modulation, modification of exposure parameters should be done. The easiest way could be to modify mAs based on the patient weight.
6. Special exposure parameters should be determined for children.
7. Proper shielding of organs
8. Limitation of scan length
9. Use of special filtration
10. Measurement of CTDI and DLP in all types of CT examinations. Compare with proposed DRLs. Repeat dose measurements in certain time intervals or when new techniques or new equipment are introduced. In the case of dose results that are higher than DRLs, corrective action should be applied.
11. Close and frequent literature research should be done to detect any scientific advances.
12. In the case of PET-CT, modification of exposure factors such as the mAs should be done. The modification should be carefully done so as to avoid the introduction of artefacts in PET images.
13. In the case of conventional CT IR procedures, lower mAs during the position of the needle if the clinical situation permits (size and position of lesion) could be used.
